# ARF‐AID: A Rapidly Inducible Protein Degradation System That Preserves Basal Endogenous Protein Levels

**DOI:** 10.1002/cpmb.124

**Published:** 2020-08-05

**Authors:** Kizhakke Mattada Sathyan, Thomas G. Scott, Michael J. Guertin

**Affiliations:** ^1^ Biochemistry and Molecular Genetics Department University of Virginia Charlottesville Virginia; ^2^ Center for Public Health Genomics University of Virginia Charlottesville Virginia; ^3^ Cancer Center University of Virginia Charlottesville Virginia

**Keywords:** ARF‐AID, auxin, auxin‐inducible degron, protein degradation

## Abstract

Inducible degron systems are widely used to specifically and rapidly deplete proteins of interest in cell lines and organisms. An advantage of inducible degradation is that the biological system under study remains intact and functional until perturbation, a feature that necessitates that the endogenous levels of the protein are maintained. However, endogenous tagging of genes with auxin‐inducible degrons (AID) can result in chronic, auxin‐independent proteasome‐mediated degradation. The ARF‐AID (auxin‐response factor–auxin‐inducible degron) system is a re‐engineered auxin‐inducible protein degradation system. The additional expression of the ARF‐PB1 domain prevents chronic, auxin‐independent degradation of AID‐tagged proteins while preserving rapid auxin‐induced degradation of tagged proteins. Here, we describe the protocol for engineering human cell lines to implement the ARF‐AID system for specific and inducible protein degradation. These methods are adaptable and can be extended from cell lines to organisms. © 2020 The Authors.

**Basic Protocol 1**: Generation of ARF‐P2A‐TIR1 progenitor cells

**Basic Protocol 2**: Designing, cloning, and testing of a gene‐specific sgRNA

**Basic Protocol 3**: Design and amplification of a homology‐directed repair construct (C‐terminal tagging)

**Alternate Protocol 1**: Design and amplification of a homology‐directed repair construct (N‐terminal tagging)

**Basic Protocol 4**: Tagging of a gene of interest with AID

**Alternate Protocol 2**: Establishment of an ARF‐AID clamp system

**Basic Protocol 5**: Testing of auxin‐mediated degradation of the AID‐tagged protein

## INTRODUCTION

A diversity of molecular tools that disrupt genes are commonly used to gain mechanistic insight into protein function, and many of the methods available today disrupt gene function by genetic knockout or RNA degradation. These methods can be universally applied to study most genes and allow us to understand the cumulative effect of gene dysregulation. The major drawbacks of these systems, however, are that the kinetics of protein depletion are slow, chronic, and often irreversible. This is problematic when studying the mechanistic function of a protein, which is most directly assessed by observing the immediate molecular and cellular response to dysregulation. Moreover, chronic gene disruption is not possible for essential genes. Small‐molecule inhibitors and temperature‐sensitive mutations are acute, rapid, and reversible means to disrupt gene function, but unique strategies are needed to target each protein of interest. In contrast, inducible degron systems allow targeted protein depletion and are rapid, reversible, and universally applicable to any protein.

Many chemical genetics approaches to targeted protein degradation utilize the exogenous expression of plant‐specific E3 ubiquitin ligase adaptor proteins in animals and cell lines. The auxin‐inducible degron (AID) system was the first heterologous system developed (Nishimura, Fukagawa, Takisawa, Kakimoto, & Kanemaki, 2009). In this system, an auxin molecule interacts with the TIR1 protein, which acts as a ubiquitin ligase adapter. This auxin‐induced interaction of AID with the SCF‐TIR1 E3 ubiquitin ligase complex causes ubiquitination and degradation of the AID‐tagged protein via the proteasome (Nishimura et al., 2009).

However, endogenously tagging genes with AID often results in unwanted chronic basal degradation in the absence of auxin. We have shown that supplementing the AID system with an additional component of the plant's native auxin signaling machinery, auxin‐response transcription factors (ARF), addresses this issue and allows the preservation of near‐endogenous expression levels of the target protein in the absence of auxin (Sathyan et al., 2019). The canonical AID system has two components: the transport inhibitor response 1 (TIR1) and auxin/indole‐3‐acetic acid (Aux/IAA or AID) proteins (Nishimura et al., 2009). In plants, there is another critical component in the auxin signal‐transduction system, the auxin‐response transcription factors. In the absence of auxin, ARF binds to the AID protein and protects it from TIR1‐mediated ubiquitination. Upon sensing auxin, TIR1 binds to and ubiquitinates the AID protein, which dissociates from ARF (Dharmasiri, Dharmasiri, Jones, & Estelle, 2003, 2005; Gray, Kepinski, Rouse, Leyser, & Estelle, 2001). Introduction of the ARF‐PB1 domain in a new version of the auxin‐inducible degron system (ARF‐AID) rescues chronic auxin‐independent degradation of AID‐tagged proteins and increases the rate of auxin‐induced degradation (Sathyan et al., 2019).

**Figure 1:**
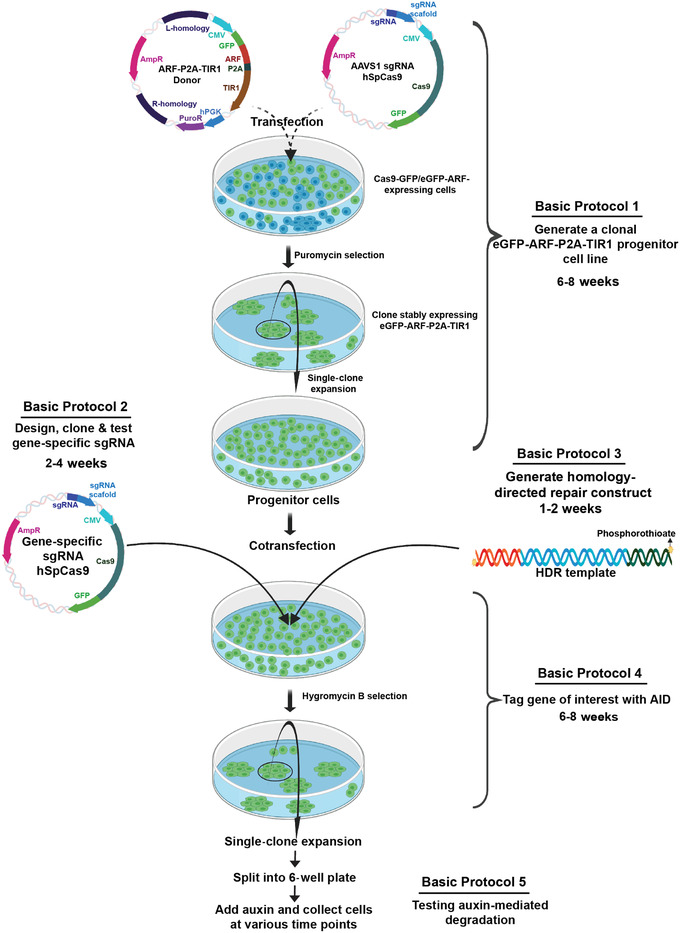
The ARF‐AID system has five distinct protocols. Basic Protocol 1 is used to generate a progenitor cell line that expresses all necessary components of the plant AID system. Basic Protocol 2 is used to test sgRNA cleavage at the gene of interest and Basic Protocol 3 to design the homology repair construct. These three protocols can be performed simultaneously. Basic Protocol 4, which incorporates all these components, describes the tagging of the protein of interest with AID in the progenitor cell line. Basic Protocol 5 describes testing for auxin‐mediated degradation of the tagged protein.

Here, we describe methods to implement the ARF‐AID system in HEK293T human embryonic kidney cells, which are easily adaptable to other cell types. We outline protocols for generating ARF‐TIR1 progenitor cells (Basic Protocol 1), designing and testing sgRNAs against the gene of interest (Basic Protocol 2), generating homology‐directed repair (HDR) constructs (Basic Protocol 3 and Alternate Protocol 1), tagging the gene of interest (Basic Protocol 4), adopting the ARF‐AID clamp system (Alternate Protocol 2), and confirming inducible protein degradation (Basic Protocol 5). An overview of the protocols is shown in Figure 1.

## STRATEGIC PLANNING

Establishing the ARF‐AID system can be time consuming if all steps are performed serially. To reduce the time required, we recommend developing the ARF‐TIR1 progenitor cells (Basic Protocol 1) in parallel with testing gene‐specific sgRNAs (Basic Protocol 2) and PCR‐amplifying the HDR constructs (Basic Protocol 3). After these protocols are complete, one can proceed with tagging the gene of interest with AID (Basic Protocol 4). TIR1‐expressing progenitor cells may already exist, developed by groups that have adopted the traditional AID system. If TIR1‐expressing progenitor cells are already available but these cells lack ARF expression, we recommend using Alternate Protocol 2 to tag the gene of interest with the AID‐ARF clamp.

Basic Protocol 1 includes a puromycin selection step after transfection. Antibiotic selection concentration varies between cell types. Before starting this protocol, we recommend plotting a curve of puromycin titration (0‐10 µg/ml final concentration) versus cell viability to determine the lowest concentration at which nearly all cells die within 5 days.

Basic Protocol 4 necessitates hygromycin selection after transfection. If you are not using HEK293T cells, then you need to titrate hygromycin (0‐500 µg/ml final concentration) and assess cell viability to determine the lowest concentration at which nearly all cells die within 7‐12 days.

Cell lysate from parental HEK293T cells is needed as a control for gDNA isolation and western blotting, so these cells should be actively cultured alongside the edited cells, or aliquots should be frozen ahead of time.

## Basic Protocol 1 GENERATION OF ARF‐P2A‐TIR1 PROGENITOR CELLS

**Figure 2:**
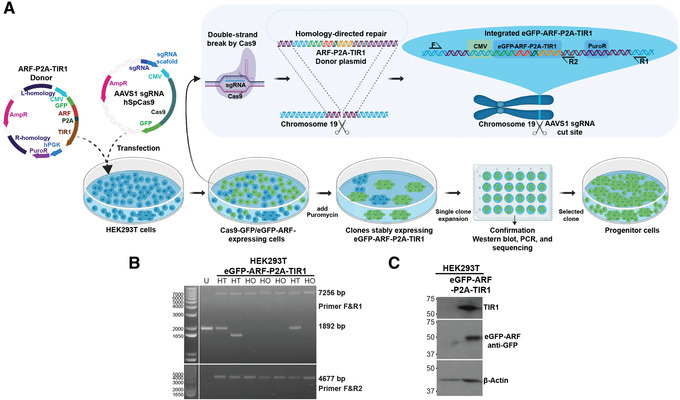
Generation of a stable progenitor cell line that expresses the components of the ARF‐AID system at a safe harbor locus. (**A**) A stepwise strategy to integrate eGFP‐ARF and TIR expressed from a CMV promoter. CRISPR is used to target the multicistronic construct into the *AAVS1* locus. Primers F and R1 generate a PCR amplicon of 7256 bp if the construct is inserted and an amplicon of 1892 bp if the construct is not inserted. R2 primer recognizes a sequence internal to the *TIR1* gene, so the primer combination F and R2 amplifies only if the construct inserts into the *AAVS1* locus. (**B**) Clones show either heterozygous or homozygous integration of the insert. Compare the bands between the unintegrated (U) and integrated clones (HT, heterozygous; HO, homozygous). The clone in the fourth lane has one allele integrated and a deletion in the other allele. (**C**) Western blotting confirms that the eGFP‐ARF and TIR1 proteins are expressed (right lane) compared to the unmodified HEK293T cells (left lane).

*NOTE*: Only one of the two plasmids listed below will be used to generate a progenitor line. As described in Background Information, first try using pMGS56 (eGFP‐ARF) to generate the progenitor cell line. If you find that the cell line is refractory to transfection or genetic editing, try using the ARF‐HA plasmid (pMGS46), which is smaller and more easily incorporated.

The first procedure for implementing the ARF‐AID system is to establish ARF‐TIR1 progenitor cells, as shown in Figure 2A. This protocol outlines the procedure of transfection, antibiotic selection, clonal cell expansion, confirmation of genetic integration, and freezing of the genetically modified progenitor cell lines.

### Materials

HEK293T cells (ATCC CRL‐3216)
HEK293T growth medium (see recipe)
PBS (Gibco 10010023)
Trypsin 0.05% (Gibco 25300054)
Opti‐MEM I reduced‐serum medium (Gibco 31985‐070)
Lipofectamine 3000 (Thermo Fisher Scientific L3000‐015)
pMGS7 (AAVS1sgRNA; Addgene no. 126582)
pMGS46 (ARF16‐PB1‐HA‐P2A‐OsTIR1; Addgene no. 126580)
pMGS56 (GFP‐ARF16‐PB1‐P2A‐OsTIR1; Addgene no. 129668), if needed
pSpCas9(BB)‐2A‐GFP (pX458; parental sgRNA cloning vector; Addgene no. 48138)
Puromycin (Gibco A11138‐03)
2× SDS sample buffer (Laemmli buffer; see recipe)
DNeasy blood and tissue genomic DNA isolation kit (Qiagen 69504)
Nuclease‐free water (Thermo Fisher Scientific AM9938)
100% ethanol
AAVS1GenomicF (primer F): 5′‐CTGCCGTCTCTCTCCTGAGT‐3′
AAVS1GenomicR (primer R1): 5′‐ACAGTTGGAGGAGAATCCACC‐3′
Internal primer (primer R2): 5′‐ATTATGATCTAGAGTCGCGGC‐3′
100% dimethyl sulfoxide (DMSO)
10 mM dNTP mix (Thermo Fisher Scientific 18427013)
Platinum Taq DNA Polymerase High Fidelity (Thermo Fisher Scientific 11304011)
6× Orange‐G Loading Dye (bioWorld 10570024‐1)
Tris/acetate/EDTA (TAE) buffer (see recipe)
SYBR Safe DNA Gel Stain (Thermo Fisher Scientific S33102)
Nonfat dry milk (Bio‐Rad 1706404)
Anti‐GFP monoclonal antibody (mAb; Sigma 11814460001)
Anti‐HA11 mAb (1:1000; BioLegend 901513)
Anti‐OsTIR1 polyclonal antibody (MBL PD048)
Anti‐β‐actin mAb (1:5000; Sigma, A1978)

Swinging‐bucket centrifuge
0.22‐µm‐pore‐size syringe filter (Millipore‐Sigma, SLGPR33RS)
Syringe (Fisher Scientific 14‐823‐16E)
Cloning cylinder (Bel‐Art SP Scienceware 378470100)
NanoDrop spectrophotometer or similar (Thermo Fisher Scientific)
0.2‐µl PCR tubes
UV transilluminator
Cryogenic storage vials (Fisher Scientific, 1050026)
Freezing container (Millipore, C1562)

Additional reagents and equipment for agarose gel electrophoresis (Voytas, 2001), SDS‐PAGE (Gallagher, 1999), mammalian cell tissue culture (Phelan & May, 2017), and western blotting (Ni, Xu, & Gallagher, 2016)

### Cotransfection of eGFP‐ARF‐P2A‐TIR1 or ARF‐HA‐P2A‐TIR1 and AAVS1 sgRNA

1 Grow HEK293T cells in HEK293T growth medium for 2 days to ∼80% confluency in a 10‐cm culture plate.
2 Remove medium from HEK293T cells and store in a 50‐ml conical tube at 4°C until step 15.
3 Wash the cells with PBS and add 0.5 ml 0.05% trypsin to the plate. Incubate the cells for 2‐3 min, rinse, and collect the cells with 10 ml medium.
4 Centrifuge the cells 5 min at 500 × *g* using a swinging‐bucket centrifuge, remove supernatant, and resuspend the cells in 10 ml fresh medium.
  Throughout this protocol, all centrifugation spins should be performed at room temperature unless otherwise stated.
5 Count cells with a hemocytometer, and seed 2.0‐3 × 10^5^ cells per well in a six‐well plate, to achieve 30%‐40% confluency per well the next day.
6 Add 125 µl Opti‐MEM I reduced‐serum medium in two 1.5‐ml tubes for each transfection; label one tube with the description of the DNA sample and the other as Lipofectamine. Add 5 µl p3000 reagent (provided with Lipofectamine 3000 reagent) to the tube labeled DNA sample. Add 5 µl Lipofectamine 3000 reagent to the tube labeled Lipofectamine
  Performing many simultaneous transfections increases the chances that you will isolate a homozygous insertion. The protocol is designed for four experimental transfections and two negative controls (parental sgRNA/Cas9 vector and untransfected) per six‐well plate.
  Transfection reagents or methods of introducing foreign DNA into cells will vary by cell line. Here, we describe the use of Lipofectamine 3000, which works efficiently for HEK293T cells.
7 Add 1 µg AAVS1 sgRNA and 1 µg of either eGFP‐ARF‐P2A‐TIR1 or ARF‐HA‐P2A‐TIR1 plasmid to the p3000 reagent mixture. Mix by pipetting up and down three times. In parallel, add 1 µg of the parental vector pX458 and the respective ARF‐TIR1 plasmid to the negative control tube.
  For HEK293T cells, we recommend a ratio of 2.5 µl Lipofectamine per microgram of DNA. If the transfection efficiency is low, increase the amounts of Lipofectamine 3000 and p3000 reagents.
8 For each transfection, transfer the Lipofectamine mixture (step 6) to the DNA/p3000 mixture (step 7) dropwise slowly. This tube now contains ∼260 µl of transfection reagents, DNA, and Opti‐MEM medium. Mix by pipetting up and down three times with the same pipette tip.
9 Incubate 15 min at room temperature, and transfer the DNA complex into the cells dropwise in a serpentine pattern to uniformly distribute the reagent across the plate.
10 Rock the plate sideways four times and front to back four times to distribute the reagent evenly over the cells—do not swirl the plate. Place cells in the incubator and incubate for 24 hr.
11 Replace the medium with fresh medium, and allow the cells to grow undisturbed for an additional 24 hr.
12 Expand each well of the six‐well plate into a 10‐cm plate with 10 ml medium as described in steps 3 and 4, and grow for an additional 24‐72 hr.
  The first time that you implement this protocol, we recommend that you grow the cells for an additional 24 hr at this point. However, if you find that there are fewer than 10 colonies total in step 17, in subsequent implementations of this step, increase the time to 72 hr.
13 Add 1 µl 10 mg/ml puromycin directly to the 10‐cm plate of cells and swirl the medium around the plate. Leave cells under selection for 3 days.
14 Replace with fresh medium containing 1 µg/ml puromycin, and continue selection for 2 days.
  After 5 total days, if there are many remaining live cells in the negative control plate, then replace with fresh medium containing puromycin and continue selection for an additional 2 days, for a total of 7 days. After 5‐7 days under selection, all the cells in the untransfected control well should be dead.
15 Transfer the conditioned medium from step 2 into a 10‐ml syringe and equip with a 0.22‐µm syringe filter. Filter the medium into a fresh 50‐ml conical tube.
  Conditioned medium is stable for 2‐3 months at 4°C.
16 Add 8 ml growth medium to 2 ml of this conditioned medium. Remove selection medium from the plates and replace with the 8:2 mixture to expand the colonies.
  Allow colonies to grow and expand. Usually, it takes 2‐3 weeks for colonies to appear after the start of puromycin selection (Fig. 2A), but the time frame is variable and dependent upon the cell line. Check the plate under a microscope and scan for colonies that appear after 2 weeks. The presence of 5‐10 times more colonies in the transfected plates compared to the control plasmid‐transfected or untransfected plates suggests successful integration of the plasmid. The control sgRNA plasmid transfected plates often contain some colonies, but far fewer.

### Picking colonies

17 Mark individual colonies on the bottom of the plate using a marker. Check for the presence of eGFP‐ARF‐P2A‐TIR1 cells using a fluorescent microscope to identify GFP‐positive colonies. Select colonies in which all the cells in the colony exhibit uniform nuclear expression of GFP. In the case of the ARF‐HA‐P2A‐TIR1‐transfected cells, you should select all colonies.
18 Carefully pick individual colonies by using 10 µl 0.05% trypsin solution and a 20‐µl micropipettor (P20 pipette) set to 20 µl. Alternatively, use cloning cylinders to pick individual colonies.
  - For hand‐picking colonies without the use of cloning cylinders: (1) hold the plate at a 45° angle to pool the medium away from the colonies; (2) use a P20 pipette set to 20 µl and aspirate ∼10 µl of trypsin solution into the pipette tip; (3) dispense a few microliters of the trypsin solution onto the colony such that a small droplet is formed between the plate and the pipette tip, taking care not to dispense so much that the droplet rolls down the plate; (4) scrape the colony with the pipette tip while the trypsin medium remains as a bridge between the plate and the tip; and (5) once the colony is dislodged, aspirate the cells into the tip and transfer of the entire colony to the 96‐well plate. Resuspend the colonies in 200 µl medium and transfer into 24‐well plates containing 1 ml medium per well.
  - Using cloning cylinders: (1) dispense silicone grease onto a glass petri dish and autoclave it along with a pair of forceps; (2) aspirate medium from the plate and wash with PBS; (3) hold the plate at a 45° angle to pool the PBS away from the colonies; (4) use forceps to pick up a cloning cylinder, dip the thicker edge into silicone grease, and place it over the colony; (5) add 20 µl of trypsin solution and incubate at 37°C until cells begin to detach; (6) resuspend the colony in 100 µl medium and transfer into a 24‐well plate containing 1 ml medium per well.
19 Wait for the cells to reach confluency before continuing, which can take between 5 and 8 days for HEK293T cells.

### Expanding cells

20 Collect cells by pipetting up and down, transfer 100 µl from a well of the 24‐well plate into a well of a fresh 24‐well plate containing 1 ml medium, and continue passaging the cells. Transfer the remaining ∼0.9 ml into a 1.5‐ml tube.
  The plate allows the expansion of the positive colonies. HEK293T cells attach loosely to the plate, so pipetting is enough to dislodge the cells. For other cell lines, collect cells by trypsinization.
21 Aliquot 100 µl of the 0.9 ml within the 1.5‐ml microcentrifuge tube into a fresh tube for genomic DNA isolation and PCR. Place cells immediately on ice.
22 Centrifuge the remaining 0.8 ml of cells for 2 min at 6000 × *g* using a fixed‐angle rotor tabletop centrifuge, and remove medium.
23 Add 100 µl 2× SDS sample buffer (Laemmli buffer) and mix thoroughly by pipetting up and down to generate cell lysate for western blotting. As a negative control, prepare cell lysate from the parental HEK293T cells.
24 Heat the lysates at 95°C for 5 min on a heating block and vortex for 10 s. Place back on the heating block for another 5 min. Briefly centrifuge the samples at 5000 × *g*, and store at −20°C.

### Screening for genomic integration at the AAVS1 locus

To screen the integration of ARF‐TIR1 at the AAVS1 locus, we use genomic PCR using primers that amplify the integrated plasmid DNA.

25 Centrifuge cells from step 21 for 2 min at 6000 × *g* using a fixed‐angle rotor tabletop centrifuge, remove medium, and either flash freeze or proceed immediately with gDNA isolation. As a negative control, also freeze cells or proceed with gDNA isolation from parental HEK293T cells.
  Use any genomic DNA isolation kit or conventional phenol‐chloroform extraction to isolate DNA from the cells. Here, we used a Qiagen genomic DNA isolation kit. The method described here is adapted from the kit's manual with minor changes (DNeasy Blood and Tissue Kit, 69504).
26 Resuspend the cells in 200 µl PBS and add 20 µl proteinase K (from kit).
27 Lyse cells by adding 200 µl Buffer AL (from kit) and mixing thoroughly by vortexing to form a homogenous lysate.
28 Incubate samples 10 min at 56°C.
29 Add 200 µl 100% ethanol and vortex.
30 Transfer the lysate into a DNeasy Mini spin column placed in a 2‐ml collection tube.
31 Spin 1 min at maximum speed in a tabletop centrifuge (10,000‐17,000 × *g*) and discard the flowthrough.
32 Place the spin column back into the collection tube and add 500 µl Buffer AW1 (from kit). Spin 1 min at 10,000‐17,000 × *g* and discard the flowthrough.
33 Place the spin column back into the collection tube, and wash by adding 500 µl Buffer AW2 (from kit) and centrifuging 1 min at 10,000‐17,000 × *g*.
34 Discard the flowthrough and centrifuge again for 2 min at maximum speed.
35 Place the spin column into a 1.5‐ml tube and add 100 µl nuclease‐free water to the center of the column. Incubate 2‐3 min at room temperature. Spin 2 min at 9000 × *g*.
  Centrifuging at 9000 × g reduces the chance of breaking off the microcentrifuge tube's lid.
36 Quantify DNA using NanoDrop spectrophotometer or similar instrument and store at −20°C.

### Genomic DNA PCR

37 Make a PCR master mix by adding all the components for the required number of reactions, using the following amounts per reaction:

**Table:** 

10 µM primer F	1.0 µl
10 µM primer R1 or R2	1.0 µl
10× High Fidelity buffer (provided with enzyme)	2.5 µl
100% DMSO	0.5 µl
10 mM dNTP mix	1.0 µl
50 mM MgSO_4_ (provided with enzyme)	1.0 µl
Platinum Taq DNA Polymerase High Fidelity	0.5 µl
Nuclease‐free water	12.5 µl
38 Aliquot 20 µl of the master mix into 0.2‐ml PCR tubes and add 5 µl of 10 ng/µl genomic DNA into each reaction mix. Use the parental HEK293T DNA as a negative control. Perform PCR using the following conditions:

**Table:** 

Initial step:	5 min	95°C	(denaturation)
30 cycles:	30 s	95°C	
	30 s	59°C	(annealing)
	7 min	68°C	(extension)
Final:	10 min	68°C	
Hold:	∞	4°C

### Gel electrophoresis

39 Prepare a 1% agarose gel with 1× TAE buffer.
40 Add 5 µl 6× Orange‐G Loading Dye directly to the PCR tubes and load onto the 1% agarose gel.
41 Run the samples for 60 min at a constant 90 V.
42 Stain the gel with SYBR Safe DNA gel stain diluted 1:10,000 with 1× TAE buffer (or 0.5 µg/ml ethidium bromide) for 10 min, and wash twice with 1× TAE for 10 min each.
43 Visualize the bands using a UV transilluminator (see Figs. 2A and B).
  The negative control produces an amplicon of 1892 base pairs (bp) with genomic F and R1 primers, whereas successful homozygous integration of the construct produces an amplicon of 7256 bp; heterozygous integration yields both amplicons. The PCR using the AAVS1GenomicF (F) and Internal Primer (R2) primers produce a 4677‐bp amplicon only in the integrated cells, and not in the negative and control cells (Figs. 2A and B).

### Confirmation of the clones by western blotting

Integration of the *ARF* and *TIR1* genes at the *AAVS1* locus does not necessarily mean the genes are expressed. Use western blotting to test whether ARF and TIR1 proteins are present in the cell.

44 Select homozygously integrated ARF/TIR1 clones from the PCR screen.
45 Thaw the frozen protein lysate (step 24), and heat again for 3‐5 min at 95°C.
46 Separate proteins by loading 10 µl of the samples on a 10% acrylamide gel, transfer the proteins onto nitrocellulose or PVDF membrane, block membrane with 7.5% nonfat dry milk, and probe with anti‐GFP or anti‐HA and anti‐TIR1 antibodies. Anti‐β‐actin (1:5000; Sigma, A1978) can be used as a loading control. A detailed western blotting protocol is available at https://currentprotocols.onlinelibrary.wiley.com/doi/10.1002/0471142727.mb1008s114 (Ni, Xu, & Gallagher, 2016). Include the parental cell lysate from step 24 as a negative control (Fig. 2C).
  After both insertion and expression are confirmed by genomic PCR and western blotting, respectively, the construct should be sequenced to confirm that mutations were not incorporated during the process.
47 Label an appropriate number of cryogenic storage vials—usually 5‐10 vials per 10‐cm culture plate. Freeze down ten 10‐cm plates, for a total of 50‐100 vials for each verified progenitor cell.
48 Remove medium from an ∼80%‐100% confluent 10‐cm plate of progenitor cells and wash with PBS. Add 0.5 ml 0.05% trypsin to the plate. Incubate the cells for 2‐3 min in the incubator, rinse, and collect cells with 10 ml of medium into a 15‐ml conical tube.
49 Count cells using a hemocytometer.
50 Add 0.5 ml of the above cells into 9.5 ml fresh medium and plate into a 10‐cm plate to maintain a backup plate. Keep this plate until the viability of the frozen cells has been tested and confirmed.
51 Centrifuge the cells from step 48 for 5 min at 500 × *g*, and remove the ∼10 ml of medium‐diluted trypsin.
52 Resuspend cells with freezing medium (prepared by adding 1 ml 100% DMSO to 9 ml HEK293T growth medium) to a final concentration of 1 × 10^6^ to 3 × 10^6^ cells/ml. Transfer 1‐ml aliquots of cells into each prelabeled freezing vial and close the vial.
53 Place the vial into a freezing container and store at least 24 hr in a −80°C freezer. Transfer the frozen vials into a liquid nitrogen tank.
54 Twenty‐four hours after storing in the liquid nitrogen, remove one vial from the liquid nitrogen and quickly thaw the cells in a 37°C water bath for 2‐4 min.
55 Transfer the cells into a 15‐ml conical tube containing 10 ml medium. Centrifuge cells 5 min at 500 × *g* and remove medium.
56 Resuspend cells in 10 ml fresh medium and plate into a 10‐cm tissue culture plate. Incubate in tissue culture incubator.
57 After 24 hr, check the viability of the newly thawed cells under a microscope.
  These frozen cell lines will serve as the progenitor cell line for tagging genes of interest with full‐length AID.

## Basic Protocol 2 DESIGN, CLONING, AND TESTING OF A GENE‐SPECIFIC sgRNA

Fusion of the AID tag to the target gene via HDR requires the specific introduction of a double strand break near the gene of interest, and this can be done using CRISPR‐Cas9. This protocol first outlines the design of sgRNAs to both the 5´ and 3´ ends of the gene of interest using Benchling (https://www.benchling.com/). Next, we explain how to clone the sgRNAs and test the efficiency of gene‐specific sgRNAs.

### Materials

BbsI restriction endonuclease (New England Biolabs R0539S)
100× BSA (New England Biolabs, B9000S)
pSpCas9(BB)‐2A‐GFP (pX458; Addgene no. 48138)
QIAquick gel extraction kit (Qiagen 28704)
QIAprep Spin miniprep kit (Qiagen 27104)
T4 DNA ligase (New England Biolabs M0202S)
T4 polynucleotide kinase (New England Biolabs M0201S)
sgRNA forward and reverse oligonucleotides, 100 µM each
Forward and reverse primers specific to the gene(s) of interest (10 µM)
Max efficiency DH5α (Thermo Fisher Scientific 18258‐012)
LB liquid medium (Fisher, BP1426) or SOC medium (Thermo Fisher Scientific, 15544034)
LB agar used to make the LB carbenicillin plates.
LB agar (Fisher, BP1425)
LB carbenicillin plates (see http://cshprotocols.cshlp.org/content/2008/8/pdb.rec11374.full)
Carbenicillin (disodium; Goldbio C‐103‐25)
Nuclease‐free water (Thermo Fisher Scientific AM9938)
LKO‐1 5′ primer 5′‐GACTATCATATGCTTACCGT‐3′
U6 promoter primer 5′‐CACAAAGATATTAGTACAAAATACG‐3′
HEK293T cells (ATCC CRL‐3216)
Opti‐MEM I reduced‐serum medium (Gibco 31985‐070)
Lipofectamine 3000 (Thermo Fisher Scientific L3000‐015)
Platinum Taq DNA Polymerase (Thermo Fisher Scientific 10966‐034)
10 mM dNTP mix (Thermo Fisher Scientific 18427013)
DMSO
Surveyor kit (IDT 706025)
6× Orange‐G Loading Dye (bioWorld 10570024‐1)
Betaine solution (Sigma, B0300)
FOXM1 sgRNA1: 5′‐GCAGGGCTCTACTGTAGCTC‐3′
FOXM1 sgRNA2: 5′‐GGGACCAGTTGATGTTGTCA‐3′
FOXM1 forward primer: 5′‐TCTGGCAGTCTCTGGATAATGAT‐3′
FOXM1 reverse primer: 5′‐GCTGATGGATCTCAGCACCACTC‐3′

Benchling software (Benchling, 2019)
Scalpel
NanoDrop spectrophotometer (Thermo Fisher Scientific) or similar
0.2‐ml PCR tubes
Thermocycler

Additional reagents and equipment for Lipofectamine transfection of sgRNA plasmid, isolation of genomic DNA, PCR, and verification of PCR amplification by agarose gel electrophoresis (see Basic Protocol 1, steps 6‐10, 26‐36, and 39‐43 and Voytas, 2001)

### Designing sgRNA using Benchling

#### Importation of target gene sequence

1 From the left navigation bar in Benchling (Benchling, 2019), click Create > CRISPR > CRISPR Guides.
2 Search for the target gene by gene ID or name, and choose the appropriate genome assembly (e.g., hg38 for human) and the transcript that is expressed in the cell line of interest.
  Optional: If there are too few nucleotides imported upstream of the start codon or downstream of the stop codon for the desired homology arm length, which is typically 50 bases, choose “Show Advanced Options” to import additional nucleotides.
3 Use default guide parameters settings or adjusted as needed; for example, the protospacer adjacent motif (PAM) sequence can be changed based on the Cas protein being used.

#### Guide selection

4 Highlight the region 25 nucleotides (nt) upstream and 25 nt downstream around the start codon (for N‐terminal tagging) or the stop codon (for C‐terminal tagging), and click “Create” to create a target sequence.
5 Benchling provides a list of guides targeting the region, along with their predicted On‐Target Scores (Doench et al., 2016) and Off‐Target Scores (Hsu et al., 2013). Among the guides with On‐Target scores >40 and Off‐Target scores >30, choose the three closest guides within 20 bases of the relevant codon. If no guides meet these criteria, choose the three guides closest to the codon, irrespective of their target scores.
6 For each of the three guides, click “Assemble” and choose pX458 as the vector into which they will be cloned.
7 For each guide, the resulting Assembly shows the predicted plasmid after cloning.
8 Return to the tab with the imported sequence, click “copy the primer list,” and paste the sequences for all generated oligonucleotides into a spreadsheet.
  The two oligonucleotides (FWD and REV) can be synthesized for downstream cloning.
  Alternatively, choose the 20‐nt target sequence without the PAM and add overhangs to clone into pX458 as follows: If the sequence does not start with a G, add a G to the 5′ end. Append 5′‐CACC‐3′ to the 5′ end of the forward sequence and 5′‐AAAC‐3′ to the 5′ end of the reverse complement of the target sequence, ensuring that the 3′ base is the complementing C nucleotide. This strategy is illustrated in Supplementary Figure S5B of Cong et al. (2013).

#### Primer design for genomic PCR at the sgRNA targeting site

After transfection, it is important to amplify the region targeted by the sgRNA to determine whether transfection of the Cas9/sgRNA vector induces mutations at the cut site. For this, you will first design a set of forward and reverse primers.

9 Open the UCSC genome browser: https://genome.ucsc.edu.
10 Select the appropriate genome, such as *Human GRCh38/hg38*.
11 Select the Blat option from the Tools drop‐down menu, paste in the sgRNA sequence, and submit.
12 View the results in the browser by clicking the *browser* action in the BLAT search results. Visually confirm the position of the sgRNA relative to genic features, such as start and stop codons.
13 Click the *View* drop‐down menu and select DNA. Modify the *Sequence Retrieval Region Option* to select 750‐bp sequence on each side of the sgRNA. This results in an ∼1500‐base region for primer selection. Click *get DNA* to retrieve the FASTA sequence file.
  Although you choose 750 bp on each side, the forward primer should be ∼250 bp upstream from the sgRNA cutting site and the reverse primer should be ∼250 bp downstream, if possible.
14 Paste the DNA sequence into Primer3 (http://bioinfo.ut.ee/primer3/; *Untergasser et al.*, *2012* *)*.
  Note that there are several tools, in addition to Primer 3, to pick appropriate primer sets, such as Primer‐BLAST (NCBI), IDT (https://www.idtdna.com/pages/tools/primerquest), or Benchling (Benchling, 2019).
15 Select appropriate parameters, or keep default settings, and submit the sequence.
  Type “750, 2” into “Targets” to include the 2 bp around the cut site in the PCR product.
  Type “500‐1000” into “Product Size Ranges” to constrain the size of the PCR product.
  To facilitate repair‐mediated mutation detection, we recommend an amplicon size of 500 bp to 1 kb with an sgRNA cut site close to the center of the amplicon.
16 Pick the best primer set, or select forward and reverse primers separately (they should have similar melting temperatures).
17 Standardize PCR conditions of the designed primers by following steps 48‐53 below and using the HEK293T genomic DNA isolated from Basic Protocol 1, step 36.
  Standardizing PCR conditions and confirming amplification can be done in parallel with steps 18‐47 below.

### Cloning the guide RNA into pSpCas9(BB)‐2A‐GFP (pX458)

#### Digestion of the vector with BbsI

18 Digest 3 µg pX458 with BbsI overnight at 37°C, using the following reaction mix:

**Table:** 

NEB buffer 2.1	5.0 µl
BbsI enzyme	1.0 µl
100× BSA	0.5 µl
pX458 plasmid	3.0 µg
Nuclease‐free water	to 50 µl

#### Gel purification of the digested plasmid

19 Add 8 µl loading dye to the mixture and load onto 1% agarose gel. Use the same amount of the undigested pX458 vector as a negative control.
20 Use a transilluminator to excise the 9266‐bp digested band using a clean scalpel, and use the QIAquick gel extraction kit (Qiagen, 28704) to isolate the digested plasmid.
  The use of the blue light dark reader transilluminator (Clare Chemical Research, USA) may reduce DNA damage during gel excision. The digested plasmid runs between the supercoiled and relaxed DNA in the undigested control sample.

#### DNA purification

The method described here is adapted from the kit manual, with minor changes (QIAquick gel extraction kit, Qiagen, 28704).

21 Add 3 vol of Buffer QG (from kit) to 1 vol of gel, considering 1 mg of gel equivalent to 1 µl. For example, add 300 µl of QG buffer for 100 mg of gel.
22 Incubate 10 min at 50°C. Mix by vortexing every 3 min to dissolve the gel. Once the gel has completely dissolved, add 10 µl 3 M sodium acetate, pH 5.0, and briefly mix by vortexing.
23 Add one gel volume of isopropanol and vortex briefly.
24 Transfer the mixture into the QIAquick column and centrifuge 1 min at 17,000 × *g*.
  If there is more dissolved gel mixture left, add to the same column and repeat step 24. Throughout this protocol, all centrifugation spins should be performed at room temperature unless otherwise stated.
25 Remove the flowthrough and place the column back into the collection tube. Add 500 µl buffer QG to the column and centrifuge 1 min at 17,000 × *g*.
26 Remove the flowthrough and place the column back into the collection tube. To wash the column, add 750 µl PE buffer and centrifuge 1 min at 17,000 × *g*.
27 Discard flowthrough, place the column back into the same tube, and centrifuge 2 min at 17,000 × *g*.
28 Place the column into a fresh clean 1.5‐ml microcentrifuge tube and add 50 µl nuclease‐free water. Incubate for 2 min and then centrifuge 2 min at 9000 × *g*.
  Centrifuging at 9000 × g reduces the chance of breaking off the lid of the microcentrifuge tube.
29 Quantify DNA using a NanoDrop spectrophotometer and store at −20°C.

#### Ligation of guide sequence into pX458 plasmid

30 Make 100 µM stock solutions of the forward and reverse strands of the sgRNA guide sequence in nuclease‐free water.
31 To phosphorylate and anneal the forward and reverse oligonucleotides of the sgRNA guide sequence in one reaction, prepare the following reaction mix (per sample):

**Table:** 

Forward (100 µM)	1 µl
Reverse (100 µM)	1 µl
T4 DNA ligase buffer	1 µl
T4 PNK	1 µl
Nuclease‐free water	6 µl
  Incubate:
  30 min at 37°C
  5 min at 95°C
  20 min at room temperature (on bench).
  The final 20‐min incubation allows the oligonucleotides to slowly anneal. The reaction mixture can then be stored at −20°C until needed.
32 Dilute the annealed sgRNA guide sequence pair 20× by adding 190 µl nuclease‐free water.
  We use 1 µl of this 500 nM dsDNA product for ligation (see next step).
33 Set up the ligation mixture below (per reaction):

**Table:** 

BbsI‐digested pX458 (step 29)	50 ng
Annealed sgRNA (step 32)	1 µl
T4 ligase buffer	1 µl
T4 DNA ligase	1 µl
Nuclease‐free water	to 10 µl
  Incubate 2 hr at room temperature or 16°C overnight. Include a no‐insert control to measure the rate of negative colonies
  Typically, we incubate 2 hr at room temperature and start the transformation. Keep the rest of the ligation mix at 16°C overnight. If the first transformation does not result in colonies, then retransform the ligated product the next day.
34 Add 2 µl of the ligated product to 20 µl chemically competent Max efficiency DH5α *E. coli*.
35 Incubate 30 min on ice, and then heat shock for 30 s at 42°C.
36 Put back on ice for 2 min.
37 Add 250 µl LB or SOC medium and incubate 1 hr at 37°C.
38 Plate the cells (272 µl) on a carbenicillin plate (stable version of ampicillin) and incubate at 37°C overnight.
39 Pick three colonies from each plate, inoculate into liquid LB medium containing carbenicillin, and incubate overnight at 37°C.
40 Pellet the bacterial culture by centrifugation for 1 min at 17,000 × *g*, and follow the steps in the QIAprep spin miniprep kit to purify plasmid DNA. Elute DNA in 50 µl nuclease‐free water.
41 Confirm the sgRNA insertion by sequencing using the LKO.1 5′ primer or the U6 promoter primer.

### Testing sgRNA using Surveyor assay

**Figure 3:**
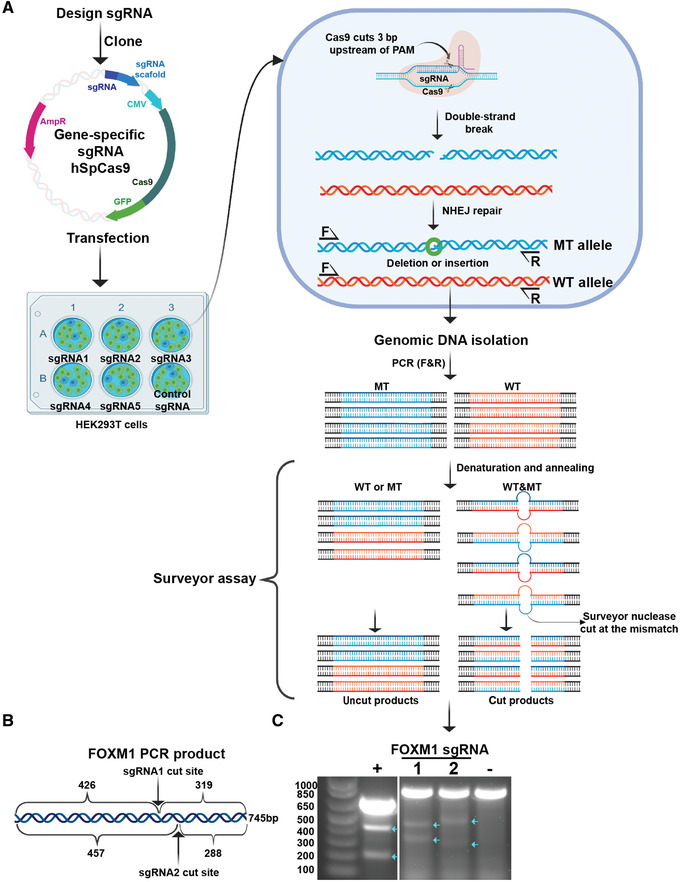
Design and testing of gene‐specific sgRNA. (**A**) A Surveyor assay is used to detect the mutation generated by nonhomologous end joining (NHEJ; green circle) at the sgRNA‐targeted sites. (**B**) Two sgRNAs were designed proximal to the 3′ end of the *FOXM1* gene. PCR primers flank the sgRNA cutting sites, and the sizes of Surveyor‐cleaved products are indicated. (**C**) The PCR products from two HEK293T cells transfected with FOXM1 sgRNA were digested with Surveyor nuclease and visualized. The positive control is provided in the Surveyor assay kit, and the negative control is a PCR product from HEK293T cells not transfected with sgRNA. Cyan arrows indicate the bands that result from heteroduplex digestion. The Surveyor assay produces three fragments: one undigested and two digested fragments.

The double‐stranded DNA breaks generated by CRISPR‐Cas9 are typically repaired by error‐prone nonhomologous repair, which results in several types of mutations, including indels. The mutated PCR products, when annealed with the wild type or other mutated PCR products, generate a mismatch proximal to the cut site. The Surveyor enzymes recognize this mismatch and cleave the heteroduplex at the site of mismatch, producing two smaller fragments that can be resolved on a gel (Fig. 3). Therefore, this assay confirms the efficiency of sgRNA in making double stranded DNA cuts at the desired site.

#### Transfection of the sgRNA construct

42 Seed 2‐3 × 10^5^ HEK293T cells per well in a six‐well plate, to achieve 30%‐40% confluency per well the next day.
43 Transfect 1 µg of the sgRNA plasmid (from step 40) with Lipofectamine 3000 reagents as described in Basic Protocol 1, steps 6‐10.
44 Replace medium with 2 ml fresh medium, and incubate for an additional 48 hr.
  GFP in the pX458 plasmid allows easy assessment of the transfection efficiency. Observe the transfected cells under a fluorescent microscope using a green filter. Higher transfection rates make it easier to assess the efficacy of the sgRNA using the Surveyor assay.
45 Remove 1 ml medium, collect the cells in the remaining 1 ml medium by pipetting up and down, and transfer into a 1.5‐ml tube.
46 Centrifuge cells 2 min at 6000 × *g* using a fixed‐angle rotor tabletop centrifuge, and remove medium.
  Cells can be stored at −20°C at this point before proceeding.
47 Proceed with genomic DNA isolation as described in Basic Protocol 1, steps 26‐36.

#### Genomic DNA PCR

Steps 48‐53 should first be performed with control HEK293T genomic DNA that is not Cas9 digested, as referenced by step 17. Once PCR conditions are optimized, proceed with PCR from both the untransfected control DNA and the experimental Cas9‐cleaved DNA from step 47.

48 Make a PCR master mix by combining the components below, adjusted for the total number of reactions:

**Table:** 

10× High Fidelity buffer (provided with enzyme)	2.5 µl
100% DMSO	0.5 µl
10 mM dNTP	1.0 µl
MgSO_4_ (provided with the enzyme)	1.0 µl
Platinum Taq DNA Polymerase High Fidelity	0.5 µl
Nuclease‐free water	12.5 µl
49 Aliquot 18 µl of this PCR master mix into 0.2‐ml PCR tubes and add 5 µl 10 ng/µl genomic DNA into each reaction mix. The parental HEK293T DNA serves as a negative control.
50 Add 1.0 µl each of forward and reverse primers (10 µM) of the corresponding gene targeted to each reaction.
51 Run PCR as follows:

**Table:** 

Initial step:	5 min	95°C	(denaturation)
30 cycles:	30 s	95°C	
	30 s	55°C‐68°C	(annealing)
	1 min	68°C	(extension)
Final:	10 min	68°C	
Hold:	∞	4°C
  Annealing and extension depend on the primers and Taq DNA polymerase used. We recommend Platinum Taq DNA polymerase or Platinum Taq DNA Polymerase High Fidelity for genomic PCR. For each primer set, it is necessary to determine the annealing temperature empirically. We find that an annealing temperature within 3° of the lowest melting temperature (T_m_) works well. Additionally, adding denaturants such as DMSO or Betaine may help to amplify GC‐rich genomic regions.
52 Add 5 µl of PCR product to 2 µl 6× Orange‐G Loading Dye and place on ice.
53 Check PCR amplification by running the sample on an agarose gel, as described in Basic Protocol 1, steps 39‐43.
  If there is only one bright PCR product, proceed with the Surveyor assay.

### Surveyor assay

Denature the PCR products from step 51 for 10 min at 95°C, and then allow to renature stepwise using the following program:

**Table:** 

10 min	95°C
1 min	85°C
1 min	75°C
1 min	65°C
1 min	55°C
1 min	45°C
1 min	35°C
1 min	25°C
∞	4°C (hold)

A ramp‐down rate of 0.3°C/s is recommended.

This step is essential because the final cycle of PCR generates homoduplexes that are not recognized by the Surveyor nuclease.

54 Set up the Surveyor nuclease reaction mix with the following (per reaction):

**Table:** 

Reannealed PCR product	20.0 µl
Surveyor Nuclease S	1.0 µl
Surveyor Enhancer S	1.0 µl
0.15 M MgCl_2_ solution	2.0 µl
55 Mix by pipetting, and incubate 60 min at 42°C in a thermocycler or water bath.
56 Stop the reaction by adding 2.4 µl of Stop Solution, and mix.
  You can safely store the reaction at −20°C for future use.
57 Follow Basic Protocol 1, steps 39‐43, for running and visualizing the samples. The ratio of the undigested band(s) to the digested band gives an estimate of relative sgRNA efficiency.
  As an example, we designed two sgRNA that efficiently target the 3′ end of the FOXM1 coding regions (Figs. 3B and C). The sequences of the sgRNAs and the primers that amplify the sgRNA target site of the FOXM1 gene are as follows:
  FOXM1 sgRNA1: 5′‐GCAGGGCTCTACTGTAGCTC‐3′;
  FOXM1 sgRNA2: 5′‐GGGACCAGTTGATGTTGTCA‐3′;
  FOXM1 forward primer: 5′‐TCTGGCAGTCTCTGGATAATGAT‐3′;
  FOXM1 reverse primer: 5′‐GCTGATGGATCTCAGCACCACTC‐3′.

## Basic Protocol 3 DESIGN AND AMPLIFICATION OF A HOMOLOGY‐DIRECTED REPAIR CONSTRUCT (C‐TERMINAL TAGGING)

**Figure 4:**
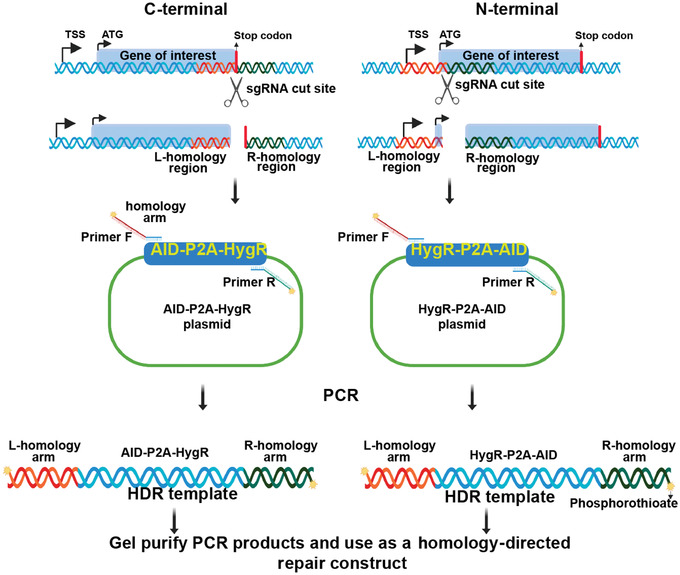
Strategy for designing the HDR constructs for the N and C termini. A 50‐nucleotide homology arm tail is added to the primers that amplify the HygR‐P2A‐AID (N) and AID‐P2A‐HygR (C) cassettes. PCR products are gel purified and used as an HDR template.

Homology‐directed repair (HDR) is the mechanism by which AID is translationally fused to the N or C terminus of the target gene. CRISPR‐Cas9 is directed to the region of interest by the sgRNA, and this complex cleaves double‐stranded DNA, which can be repaired by nonhomologous end joining or homologous recombination/repair. For C‐terminal fusions, the repair construct consists of AID separated from the hygromycin‐resistance gene (*HygR*) by a porcine teschovirus‐1 ribosomal skipping sequence (P2A; Kim et al., 2011; Fig. 4). In the N‐terminal repair construct, the order is reversed: HygR‐P2A‐AID (Fig. 4). In both cases, the AID is separated from the protein of interest by adding a linker sequence of 6‐9 amino acids (2‐3× GGS).

The HDR construct can be PCR products or cloned into a plasmid. So, the first step in the HDR construct synthesis is designing primers to amplify the HDR template either for cloning into a plasmid (Elion, Marina, & Yu, 2007) or for direct transfection of the PCR product. We recommend direct transfection of the gel‐purified PCR product. Different parameters that are outlined in the Commentary section are taken into consideration when designing primers for N‐terminal or C‐terminal tagging depending on the site of sgRNA targeting. Follow Basic Protocol 3 for C‐terminal tagging and Alternate Protocol 1 for N‐terminal tagging.

### Materials

pMGS54 (AID‐P2A‐Hygromycin; Addgene, no. 126583)
Platinum Taq DNA Polymerase High Fidelity (Thermo Fisher Scientific 11304011)
10 mM dNTP mix (Thermo Fisher Scientific 18427013)
100% DMSO
Nuclease-free water (Thermo Fisher Scientific AM9938)
NanoDrop spectrophotometer (Thermo Fisher Scientific) or similar

Additional reagents and equipment for agarose gel electrophoresis and gel purification of PCR products (see Basic Protocol 1, steps 18‐28, and Voytas, 2001) and constructing recombinant DNA molecules by PCR (Elion et al., 2007)

### Tagging with upstream homology arm (coding‐strand primer design)

1 The upstream homology arm begins 50 bases upstream of the cut site, and the last base is the nucleotide immediately upstream of the first stop codon base. If the cut site is upstream of the stop codon, the coding nucleotides downstream of the cut site will need to be modified at the codon wobble bases.
2 Append 5′‐GGTGGATCTGGAGGTTCAGGTGGCAGTGTCGAGCTGAATCT‐3′ to the 3′ end of the upstream homology arm for C‐terminal tagging using the insert from pMGS54. This sequence contains a flexible linker region upstream of the AID coding sequence.

### Tagging with downstream homology arm (template‐strand primer design)

3 The downstream homology arm begins 50 bases downstream of the stop codon and extends to the cut site. If the cut site is in the middle of a codon, ensure the AID‐P2A‐HygR cassette is in frame with the protein by adding extra nucleotides.
  Alternatively, if the cut site is downstream of the stop codon, the homology arm can extend to the nucleotide immediately downstream of the stop codon. This will include the full 3′ UTR, but may decrease the efficiency of HDR. Make necessary mutations in the 3′ UTR to ensure that the sgRNA will not recognize the product after repair.
4 Append 5′‐TCAGTTAGCCTCCCCCATCTC‐3′ to the 3′ end of the downstream homology arm for C‐terminal tagging using the insert from pMGS54. This sequence contains the template strand of the *HygR* coding sequence.
  PCR with the above primers and pMGS54 as a template results in an amplicon of 1791 bp plus the length of the homology arms.
5 Add phosphorothioate moieties to the first two 5′ nucleotides of both upstream and downstream primers.
  Important: Confirm that the AID‐P2A‐HygR cassette is in frame with the protein and that the sgRNA will not recognize the product after repair by in silico PCR using SnapGene or any other program.

### PCR amplification of the HDR template

6 Synthesize the designed primers including the phosphorothioate bonds at the 5´ end.
7 To amplify the HDR template, prepare the following PCR mix using the primers and Platinum Taq DNA Polymerase High Fidelity (per sample):

**Table:** 

Plasmid DNA (pMGS54 or 58)	50.0 ng
10 µM Primer F	1.0 µl
10 µM Primer R	1.0 µl
10× High Fidelity buffer (provided with enzyme)	5.0 µl
100% DMSO	0.5 µl
10 mM DNTP	2.0 µl
MgSO_4_ (provided with the enzyme)	2.0 µl
Platinum Taq DNA Polymerase High Fidelity	0.5 µl
Nuclease‐free water	to 50 µl
  Perform PCR amplification using the following conditions:

**Table:** 

Initial step:	5 min	95°C	(denaturation)
30 cycles:	30 s	95°C	
	30 s	60°C	(annealing)
	1 min	68°C	(extension)
Final:	10 min	68°C	
Hold:	∞	4°C	(extension).
  We perform several 50‐µl reactions (typically four to eight) and gel purify the PCR products.
  Removing any remaining primer from the PCR product is important, as primers may interfere with homologous recombination. The primers are very long, so conventional PCR cleanup kits will not remove them efficiently, and they then can reduce the HDR efficiency by binding to the cut site. Therefore, always gel purify the PCR products.

### Agarose gel purification of the template

8 Run the PCR products on an agarose gel and cut out the repair construct band at ∼1800 bp as described in Basic Protocol 2, steps 19 and 20.
9 Combine all the gel slices into one tube and purify the DNA using the Qiagen gel purification kit as described in Basic Protocol 2, steps 21‐28.
10 Quantify the repair construct band using a NanoDrop spectrophotometer and store at −20°C.

## Alternate Protocol 1 DESIGN AND AMPLIFICATION OF A HOMOLOGY‐DIRECTED REPAIR CONSTRUCT (N‐TERMINAL TAGGING)

### Materials

pMGS58 (Hygromycin‐P2A‐AID; Addgene no. 135311)

Additional reagents and equipment for constructing recombinant DNA molecules by PCR (Elion et al., 2007)

### Tagging with upstream homology arm (coding‐strand primer design)

1 The upstream homology arm starts 50 bases upstream of the start codon and ends at the cut site. If the cut site is in the middle of a codon, ensure the HygR‐P2A‐AID cassette is in frame with the protein by adding extra nucleotides.
  Alternatively, if the cut site is upstream of the start codon, the homology arm can extend to the nucleotide immediately upstream of the start codon. This will include the full 5′ UTR, but may decrease the efficiency of HDR. Make necessary mutations in the 5′ UTR to ensure that the sgRNA will not recognize the product after repair.
2 Append 5′‐ATGAAAAAGCCTGAACTCACCG‐3′ to the 3′ end of the upstream homology arm for N‐terminal tagging using the insert from pMGS58. This sequence contains the beginning of the *HygR* coding sequence.

### Tagging with downstream homology arm (template‐strand primer design)

3 The downstream homology arm begins 50 bases downstream of the cut site, and the last base is the nucleotide immediately downstream of the last start codon base. If the cut site is downstream of the start codon, the coding nucleotides upstream of the cut site will need to be modified at the codon wobble bases.
4 Append 5′‐CCCACCTGAACCTCCAGATC‐3′ to the 3′ end of the downstream homology arm for N‐terminal tagging using the insert from pMGS58. This sequence is complementary to the coding sequence of a flexible linker sequence following the end of the AID coding sequence in the plasmid.
  To functionally separate the protein of interest from AID, a linker of 9 amino acids is added at the C‐terminus of the AID in the pMGS58 plasmid. The provided primer (below) amplifies both the linker and the AID. If any other template is used for generating the tag, be sure to add the linker amino acid sequence. The PCR with the above primers using pMGS58 as a template produces an amplicon of 1815 bp plus the length of the homology arms.
5 Continue with steps 5‐10 of Basic Protocol 2.
  Important: Confirm that the HygR‐P2A‐AID cassette is in frame with the protein and that the sgRNA will not recognize the product after repair by in silico PCR using SnapGene or any other program.

## Basic Protocol 4 TAGGING OF A GENE OF INTEREST WITH AID

**Figure 5:**
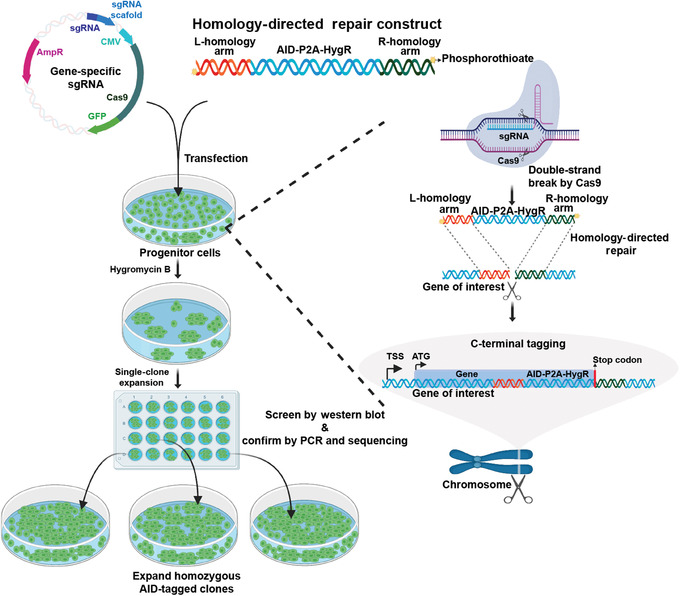
HDR‐mediated integration of AID tag at the 3′ end of the gene of interest using CRISPR‐Cas9. HEK293T‐eGFP‐ARF‐P2A‐TIR1 cells are cotransfected with an sgRNA and a PCR amplified repair construct. The cells are selected with hygromycin B, and the clones are screened for integration using western blotting, PCR, and sequencing.

The next step in adopting the ARF‐AID system is to tag the gene of interest with full‐length AID. The three main steps in the tagging of a gene with AID are (1) cotransfection of a gene‐specific sgRNA and HDR template into ARF‐TIR1 progenitor cells; (2) selecting tagged clones with hygromycin B; and (3) clonal expansion and confirmation of tagging. A general outline of these steps is illustrated in Figure 5.

### Materials

ARF‐TIR1 progenitor cells (Basic Protocol 1, step 53)
Hygromycin B (Thermo Fisher Scientific, 10687010)

Additional reagents and equipment for collection of conditioned medium, cotransfection of sgRNA plasmid and HDR template, picking colonies, expanding cells, and freezing cells (Basic Protocol 1, steps 2, 6‐10, 15, 17‐24, and 47‐57), genomic DNA isolation with QIAquick gel extraction kit (Basic Protocol 2, steps 19‐29), and western blotting (Ni, Xu, & Gallagher, 2016)

1 Grow the ARF‐TIR1 progenitor cells, split into a six‐well plate with 2‐3 × 10^5^ cells seeded per well, and collect conditioned medium as described in steps 2 and 15 of Basic Protocol 1.
2 Cotransfect 1 µg of gene‐specific sgRNA plasmid and 400 ng of double‐stranded HDR template PCR product to the cells as described in Basic Protocol 1, steps 6‐10. Use 5‐7.5 µl Lipofectamine reagent for transfection.
  The parental pX458 cotransfected with HDR PCR product is used as a transfection control, and we recommend keeping an untransfected control cell well. Transfect multiple (at least four) wells to obtain a sufficient number of colonies.
3 Replace the medium with fresh medium, and allow the cells to grow undisturbed for an additional 24 hr.
4 Expand each well into a 10‐cm plate and incubate for 24‐72 hr.
  The first time that you implement this protocol, we recommend that you grow the cells for an additional 24 hr. However, if you find that there are fewer than 10 colonies total in step 8, in subsequent implementations of this step, increase the time to 72 hr.
  Expanding cells into 10‐cm plates ensures that the cells are sufficiently sparse to allow colonies to form in isolation.
5 Add 20 µl hygromycin B to a final concentration of 100 µg/ml and swirl the medium. Alternatively, add an empirically determined concentration as described in Strategic Planning.
6 Replace with fresh medium containing 100 µg/ml hygromycin B and continue selection for 3 days.
7 Monitor the cells daily until all the cells in the control plate are dead (7‐12 days), and then replace the medium with a mixture of 8 ml growth medium and 2 ml conditioned medium.
8 Colonies will appear in the plates after 2 or 3 weeks.
9 Pick colonies and expand cells as described in Basic Protocol 1, steps 17‐24, and screen for tagged clones by western blotting and gDNA PCR.
  In order to quantify the relative levels of the protein, use antibodies specific for the protein of interest, as opposed to α‐AID antibodies. Protein‐specific antibodies are necessary to quantify the tagged protein levels relative to the progenitor cells in the subsequent protocol. Successful tagging results in an increase in protein size of 24 kDa. Heterozygous integration will contain bands reflecting both the native and tagged proteins. Nonhomologous end repair is error prone and may result in an unexpected size shift that is not ∼24 kDa. Here, you are only determining whether the clonal cell lines exhibit an appropriate size shift and whether they are homozygous. Basic Protocol 5 is used to determine the relative expression level of the tagged protein compared to the progenitor. Confirm the integration and reading frame of the integrated AID‐P2A‐HygR or HygR‐P2A‐AID by PCR and sequencing; see below.
10 Select colonies from the western blotting experiment and perform genomic DNA PCR using the same set of primers used for testing sgRNA efficiency according to Basic Protocol 2, steps 48‐51, except with the PCR extension time increased to 4 min to amplify the insert.
11 Run the whole PCR product on 1% agarose gel, excise the band using a clean scalpel, and use the Qiagen gel purification kit to isolate genomic DNA (follow Basic Protocol 2, steps 19‐29). Sequence the purified PCR product with the forward primer to confirm the reading frame.
12 Freeze the cell lines as described in steps 47‐57 of Basic Protocol 1.

## Alternate Protocol 2 ESTABLISHMENT OF AN AID‐ARF CLAMP SYSTEM

**Figure 6:**
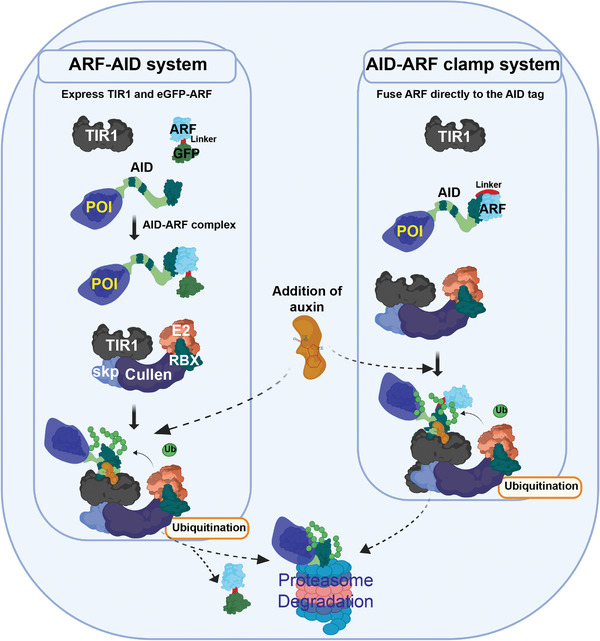
An overview of the components and their roles in engineered ARF‐AID systems. The ARF‐AID system (left) differs from traditional AID systems due to the presence of the ARF‐PB1 domain, which binds to the AID tag and prevents auxin‐independent AID degradation. The AID‐ARF clamp system (right) fuses the ARF‐PB1 domain to the AID tag, which also protects AID from auxin‐independent degradation. An advantage of the clamp system is that previously generated TIR1‐expressing cells and animals can be used as the progenitors for protein tagging. In both systems, auxin facilitates interaction between AID and TIR1 to mediate rapid degradation.

**Figure 7:**
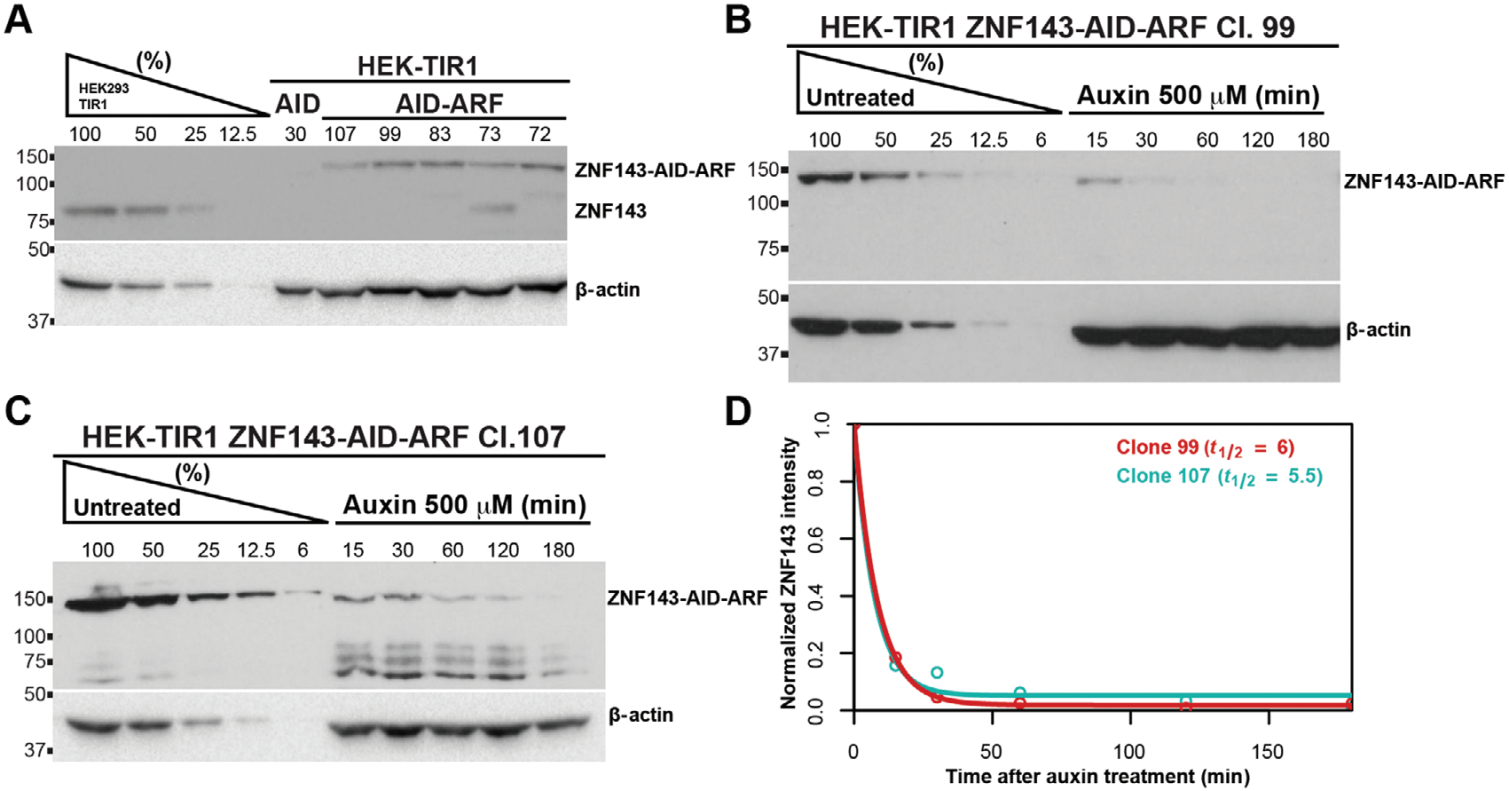
The AID‐ARF clamp system facilitates the rapid auxin‐inducible degradation of the AID‐ARF‐tagged proteins. (**A**) ZNF143‐AID‐ARF is expressed at levels comparable to those in the parental TIR1‐expressing cells. (**B** and **C**) Two independent ZNF143‐AID‐ARF clones show rapid ZNF143 degradation upon auxin treatment. (**D**) Protein half‐lives of the clones are between 5 and 6 min.

All AID systems necessitate the expression of TIR1, so there are many progenitor cell lines and organisms already available that express TIR1 (Holland, Fachinetti, Han, & Cleveland, 2012; Li, Prasanna, Salo, Vattulainen, & Ikonen, 2019; Natsume, Kiyomitsu, Saga, & Kanemaki, 2016; Nishimura et al., 2009; Zhang, Ward, Cheng, & Dernburg, 2015). In an effort to repurpose these cell lines and organisms but alleviate chronic degradation of target proteins, one can fuse the AID tag with ARF using a flexible linker to create the AID‐ARF clamp (Fig. 6). This protocol covers tagging of the C‐terminus of the protein of interest with the AID‐ARF clamp using Addgene plasmid no. 138174. Here, we describe the procedure to tag *ZNF143* at the C‐terminus with the AID‐ARF fusion protein (Fig. 7) using the sgRNA and the donor primers given in the Materials list.

### Materials

pMGS59 (AID‐ARF‐P2A‐Hygromycin; Addgene no. 138174)
pMK232 (CMV‐OsTIR1‐PURO; Addgene no. 72834), optional
pMGS7 (AAVS1sgRNA; Addgene no. 126582), optional
*ZNF143* C‐terminal targeting sgRNA: 5′‐GAGGATTAATCATCCAACCC‐3′
*ZNF143* C‐terminal HDR construct PCR primers:
Forward: 5′‐A*A*GAAGCCATCAGAATAGCGTCTAGAATCCAACAAGGAGAAACGCCAGGGCTTGACGACGGTGGATCTGGAGGTTCAGGTGGCAGTGTCGAGCTGAATCT‐3′
Reverse: 5′‐*A*GACTCCTTCTGCTTTATTGCTCCATTGTTCTGAGGATTAATCATCCAATCAGTTAGCCTCCCCCATCTC‐3′ (where * denotes phosphorothioate bond modifications)

Additional reagents and equipment for Basic Protocols 1‐4 as needed (see step 1)

1 If TIR1‐expressing progenitor cells are available, proceed to step 2 and directly tag the protein of interest with the AID‐ARF clamp using pMGS59. Alternatively, you need to generate a TIR1‐expressing progenitor cell using the TIR1 plasmid developed by the Kanemaki lab (Natsume et al., 2016; Addgene no. 72834) and the sgRNA that targets the *AAVS1* locus (Addgene no. 126582). Follow Basic Protocol 1, steps 1‐57, to generate progenitor cells using this construct.
2 To tag the protein of interest with the ARF‐AID clamp in the TIR1 progenitor cells, follow Basic Protocols 2, 3, and 4. The only differences are the progenitor cells (TIR1 as opposed to ARF/TIR1) and the HDR template. Use the AID‐ARF‐P2A‐Hygromycin plasmid (Addgene no. 138174) to generate the HDR template.

## Basic Protocol 5 TESTING OF AUXIN‐MEDIATED DEGRADATION OF THE AID‐TAGGED PROTEIN

Before using the AID‐tagged cell lines to study the effect of acute protein depletion, use the following protocol to quantify the protein expression of the AID‐tagged protein in comparison with the parental cells and measure the degradation rate upon auxin treatment.

### Materials

AID‐tagged clones (Basic Protocol 4)
Progenitor cells (Basic Protocol 1, step 53)
Auxin (3‐indoleacetic acid, sodium salt; Abcam ab146403)
ImageJ software

### Quantify the expression level of the AID‐tagged protein

**Figure 8:**
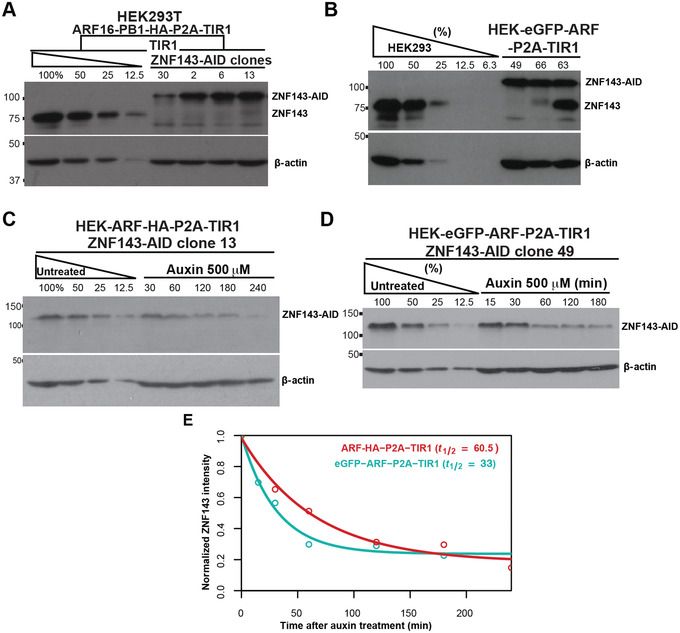
The ARF‐AID system preserves the endogenous expression levels of AID‐tagged proteins and facilitates auxin‐inducible degradation. Tagging ZNF143 with AID in both an ARF‐HA‐P2A‐TIR1 (**A**) and a eGFP‐ARF‐P2A‐TIR1 (**B**) progenitor line preserves comparable expression levels compared to the progenitor lines. (**C** and **D**) Both ZNF143‐AID‐tagged ARF‐HA‐P2A‐TIR1 and eGFP‐ARF‐P2A‐TIR1 cell lines facilitate auxin‐inducible ZNF143‐AID degradation with (**E**) protein half‐lives of 60 and 33 min, respectively.

1 Thaw AID‐tagged clones and progenitor cells and culture as described in Basic Protocol 1, steps 54‐56. Wait ∼3 days for the cells to reach >80% confluency.
2 Seed ∼7‐8 × 10^5^ of each positive AID‐tagged clonal cell line into a well of a six‐well plate. The number of six‐well plates is determined by the number of sequence‐verified clones from Basic Protocol 4. Include a single well for the ARF‐TIR1 progenitor line without an AID‐tagged protein of interest. Wait 24 hr, which should result in ∼75% confluent cells in each well.
  The remaining cells can be maintained and passaged, as they will be needed for step 15 below.
3 Remove 1 ml medium from each well, collect all the cells by pipetting up and down in the remaining medium, and transfer the cells to a 1.5‐ml microcentrifuge tube. Put the cells on ice.
4 Centrifuge cells immediately after collection using a fixed‐angle rotor tabletop centrifuge for 2 min at 6000 × *g*, 4°C, and carefully remove medium by using a pipette.
5 Add 200 µl 2× SDS sample buffer directly into the pellet and pipette up and down several times. The lysate will become highly viscous.
  Alternatively, add the 2× SDS sample buffer into the plate after removing the medium to directly lyse the cells. Collect the lysate with a pipette.
6 Heat‐denature proteins for 5 min at 95°C, vortex 20 s, and denature again for another 5 min. Store the lysate at −20°C.
7 Check the expression level of the tagged protein by western blotting (Fig. 7A; Fig. 8A and B). Serially dilute the lysate of the untagged progenitor cell line that expresses ARF and TIR1 to ensure that the query bands of the western are within the linear range of the assay and to compare the level of expression in the tagged clones. Load the serial dilutions of the progenitor control lysate followed by all the AID‐tagged clone cell lysates. Continue with western blotting using antibodies directed against the AID‐tagged protein (Ni, Xu, & Gallagher, 2016); use actin as a loading control.

8 Measure the density of both the protein of interest and corresponding control (actin) bands using ImageJ.
9 Divide the signal density of the protein of interest by the signal density of the control actin signal to account for loading differences and to obtain the relative intensity of your protein.
10 For the progenitor cell lysate serial dilution, the amount of protein in each lane is known relative to the undiluted sample. Plot the densitometry intensities against the known fraction of cells in the standard curve for both the actin bands and the bands for the protein of interest. Check that the standard curve intensities are linearly correlated to the fraction of cells loaded (Guertin & Lis, 2010). Very intense and very modest bands are most likely to fall outside the liner standard curve. The linear range of the assay is determined by the linear portion of the standard curve.
11 Fit a linear regression to the standard curve.
  Points outside the linear range of the assay should not be included in the model.
  In Microsoft Excel, you can plot a scatter plot of the intensities (y axis) and fraction of cells loaded (x axis), add a linear trendline, and display the equation on the chart.
12 Plug the protein of interest densitometry intensity into this equation as the *y* variable and solve for *x*. This value represents the relative intensity of the protein of interest compared to the progenitor cell line. The measured intensities represent the quantitative range of the assay, and one cannot use the regression formula to extrapolate beyond this range.

### Measure the degradation rate of the AID‐tagged proteins

13 Prepare 50 mM auxin in water, divide into 500‐µl aliquots, and store at −20°C.
  Auxin is stable for several months at −20°C. Use a fresh aliquot each time and do not refreeze.
14 Label the six wells of the plate: no treatment, 15 min, 30 min, 1 hr, 2 hr, and 3 hr.
15 Choose AID‐tagged HEK293T cell lines that express the tagged protein at the most comparable level to the progenitor cell line and seed ∼7‐8 × 10^5^ cells in each well of a six‐well plate. Wait 24 hr, which should result in ∼75% confluent cells in each well.
  These cells can be thawed fresh, as described in step 1, or the cells that are being passaged and maintained (see note following step 2) can be used.
16 Add a final concentration of 500 µM auxin dropwise to the medium all over the plate and mix by moving the plate forward, backward, and sideways. Do not swirl the plate.
17 Collect cells at regular intervals starting from no auxin treatment. Remove 1 ml of medium from each well and collect cells by pipetting up and down in the remaining medium and transfer the cells to a 1.5‐ml microcentrifuge tube. Put the cells on ice.
  Initially, we recommend time intervals of 15 min, 30 min, 1 hr, 2 hr, and 3 hr, but depending on the degradation rate, subsequent time points should be empirically determined.
  Keeping cells on ice significantly reduces further degradation of the tagged protein.
18 Centrifuge cells immediately after collection using a fixed‐angle rotor tabletop centrifuge for 2 min at 6000 × *g*, 4°C, and carefully remove medium by using a pipette.
  Centrifugation at 4°C significantly reduces further degradation of the tagged protein.
19 Add 200 µl 2× SDS sample buffer directly into the pellet and pipette up and down several times. The lysate becomes highly viscous.
  Alternatively, add the 2× SDS sample buffer into the plate after removing the medium to directly lyse the cells. Collect the lysate with a pipette.
20 Heat‐denature proteins 5 min at 95°C, vortex 20 s, and denature again for another 5 min. Store the lysate at −20°C.
21 Check the auxin‐induced degradation of the tagged protein by western blotting (Figs. 7B‐D and 8C‐E). Serially dilute the untreated lysate to ensure that the query bands of the western are within the linear range of the assay. Load the serial dilution of the untreated AID‐tagged control lysate, and also include the treated cell lysate. Continue with western blotting using antibodies directed against the AID‐tagged protein.
  The degradation of the AID‐tagged proteins starts immediately after the addition of auxin.
22 Measure the density of both the AID‐tagged protein and corresponding control (actin) bands using ImageJ. Account for loading differences by dividing the signal for the AID‐tagged protein by the signal for the actin control. Normalize the auxin‐treated samples to the zero/no treatment timepoint such that the signal at time 0 is equal to 1.
23 Determine the rate of degradation by plotting the intensity of the AID‐tagged protein bands (Figs. 7D and 8E) and fit the data using the following equation (see Internet Resources), where *y*(*t*) is the intensity of the protein time point *t*; *y*(0) is the initial relative intensity of the protein band, usually ∼1.0; *y*_∞_ is the y value that is asymptotically approaching at the infinite time point; *k* is the rate constant; and *t* is the time point:
$$y\left(t\right)=\left(y_{0}-y_{\infty }\right)e^{-kt}+y_{\infty }$$

## REAGENTS AND SOLUTIONS

### HEK293T growth medium (565 ml)

500 ml DMEM (Gibco 11965‐092)
5 ml 100 mM sodium pyruvate (Gibco 11360070)
5 ml 100× l‐glutamine (Gibco 25030081)
50 ml fetal bovine serum (VWR 89510186)
5 ml Pen/Strep (Gibco 15140122)

### SDS buffer (Laemmli buffer), 5×

312.5 mM Tris·Cl, pH 6.8
10% SDS
50% (w/v) glycerol
0.05% bromophenol blue
Store at room temperature
Dilute 5× SDS buffer with water to 2×, and add 50 μl 2‐mercaptoethanol per ml of 2× SDS sample buffer prior to use. The 2× SDS sample buffer with 2‐mercaptoethanol can be stored at −20°C.

### Tris/acetate/EDTA (TAE) buffer, pH 7.2, 50×

2 M Tris base
1 M sodium acetate
50 mM EDTA
Adjust pH to 7.2 with acetic acid and store at room temperature.
Prepare 1× working solution by diluting in double‐distilled water.

## COMMENTARY

### Background Information

A common strategy to directly manipulate protein stability is to induce interaction with a ubiquitin ligase complex, which leads to polyubiquitination and proteasomal degradation (Sakamoto et al., 2001; Schapira, Calabrese, Bullock, & Crews, 2019; Schneekloth, Pucheault, Tae, & Crews, 2008). Proteolysis‐targeting chimeras (PROTACs) are heterobifunctional molecules that promote proximity‐mediated polyubiquitination. PROTACs are composed of a moiety that binds to an E3 ubiquitin ligase, such as von Hippel‐Lindau (VHL) or cereblon (CRBN), and a small molecule that directly interacts with the protein of interest (Bondeson et al., 2015; Lu et al., 2015; Schapira et al., 2019; Winter et al., 2015). This strategy requires a chemical probe for the protein of interest as starting material, and developing PROTACs for each target is time consuming and requires medicinal chemistry expertise.

The dTAG system provides a more universal system to specifically target proteins of interest for rapid and inducible ubiquitin‐mediated degradation. In the dTAG system, the protein of interest is fused to a mutant human mTOR signaling protein, FKBP^F36V^, and a single bifunctional molecule (dTAG‐13) promotes proteasome targeting (Nabet et al., 2018). This system is simple and requires only one genetic manipulation in order to tag the protein with FKBP^F36V^. However, the degradation rate using this system varies depending on the cell type (Li et al., 2019). Additionally, the amount of the dTAG‐13 molecule must be titered based on protein levels in order to avoid saturating each end of the molecule independently and not providing a link between the target and the ubiquitin ligase (Nabet et al., 2018; Li et al., 2019).

The auxin‐inducible degron (AID) system introduced the concept of utilizing the plant auxin‐sensing pathway to develop a heterologous degron system in animal cells (Nishimura et al., 2009). This was followed by the development of the jasmonate‐inducible degron (JID) system. In that system, in the presence of jasmonate‐isoleucine, proteins tagged with the JAZ degron interact with the F‐box containing COI1 and are subsequently degraded (Brosh et al., 2016). Recently, another auxin‐sensing F‐box protein, *Arabidopsis thaliana* AFB2 (AtAFB2), has been developed as a promising new degron system (Li et al., 2019). We look forward to mixing and matching the components of newly developed AID systems in order to further refine these tools.

Of the direct protein degradation technologies, the auxin‐inducible degron system is the most robust and most widely used (Lambrus, Moyer, & Holland, 2018). Unlike the dTAG system, the two‐component AID system allows tissue‐specific degradation of the AID‐tagged protein by controlling tissue‐specific expression of TIR1. Stable expression of *ARF* and *TIR1* ensures efficient auxin‐inducible degradation of the AID‐tagged proteins. Integrating these genes at a safe‐harbor genetic locus (Fig. 2) allows ARF‐PB1 and TIR1 to be stably expressed and resistant to epigenetic silencing. Virus‐mediated integration of the constructs at random genetic loci, on the other hand, may lead to variable and unstable expression of ARF and TIR1. We incorporate *ARF* and *TIR1* (eGFP‐ARF‐P2A‐TIR1 or ARF‐HA‐P2A‐TIR1) into the human *AAVS1* safe‐harbor locus. Redesigning the eGFP‐ARF‐P2A‐TIR1 plasmid with *ROSA26*‐specific homology arms and using a mouse *ROSA26*‐specific sgRNA (Chu et al., 2016) will allow integration into mouse cells. For cells from other organisms, users can design a sgRNA to a safe‐harbor locus and design right and left homology arms to flank the eGFP‐ARF‐P2A‐TIR1 or ARF‐HA‐P2A‐TIR1 construct. The integration plasmids have homology arms ∼800 nt in length. Shorter homology arms, of as few as 30 nt, also permit efficient HDR and have the advantage of increased transfection efficiency (Paix et al., 2017). We recommend generating a clonal progenitor cell line that expresses ARF and TIR1, and then using this progenitor to tag proteins of interest. We provide human‐specific codon optimized constructs, but codon optimization is recommended for expression in other organisms.

Simultaneous expression of ARF and TIR1 driven by a robust common promoter ensures high expression of these proteins compared to most cellular proteins (Fig. 2). We generated two multicistronic plasmids that express both ARF and TIR1 driven by a CMV promoter (Sathyan et al., 2019). A P2A ribosome‐skipping site separates these two polypeptides during translation (Fig. 2A). In our original work, we used a CMV‐driven GFP‐ARF to rescue the chronic degradation of AID‐tagged proteins (Sathyan et al., 2019). In that context, we found that the rescued AID‐tagged proteins degraded faster when treated with auxin (Sathyan et al., 2019). As opposed to rescuing protein levels, we recommend preserving levels by generating a progenitor cell line that coexpresses TIR1 and ARF. Expression of either ARF‐HA or GFP‐ARF‐P2A‐TIR1 mitigate auxin‐independent degradation (Fig. 8A and B). Expression of GFP‐ARF promotes more rapid degradation kinetics than expression of ARF‐HA (Fig. 8C‐E), so we recommend using the GFP‐ARF construct for the progenitor cell line. The ARF‐HA construct is smaller and thus more amenable to genetic insertion if the cell line is refractory to genetic editing.

Here, we introduced the ARF‐AID clamp system by C‐terminal tagging ZNF143 with AID‐ARF clamp and using a canonical TIR1 expressing progenitor cell line. Similar to the ARF‐AID system, the AID‐ARF clamp preserves near‐endogenous protein expression (Fig. 7A). Moreover, the AID‐ARF‐clamp‐tagged ZNF143 protein degraded rapidly upon auxin treatment (Fig. 7B and C). Both ZNF143‐AID‐ARF clones tested have an average half‐life of between 5 and 6 min upon auxin treatment (Fig. 7D). For N‐terminus ARF‐AID clamp tagging, the order of the AID and ARF fusion and linker properties need to be empirically determined. In the future, we look forward to testing whether AID‐ARF‐clamp‐tagged proteins consistently degrade more rapidly than tagged proteins from the canonical AID and multicistronic ARF‐AID systems.

### Critical Parameters

#### Subcellular localization of ARF

ARF prevents chronic degradation of the AID‐tagged proteins by direct interaction with AID (Sathyan et al., 2019). Therefore, it is important that ARF and the AID‐tagged protein be localized to the same subcellular region. The ARF‐TIR1 plasmids described here are designed for proteins localized in the nucleus, so a nuclear localization signal (NLS) sequence is fused to the ARF‐PB1 domain (Sathyan et al., 2019). In order to adopt the system to degrade cytoplasmic or uniformly distributed proteins, one should replace the NLS with a nuclear export signal or delete the NLS. If the plasmid is modified to localize ARF into cytoplasm or uniformly, you will need to confirm the localization of ARF using either GFP fluorescence or immunofluorescence against HA, depending on the ARF tag.

#### Designing sgRNAs

Consider two parameters in choosing an sgRNA: the distance from the desired homologous repair site and the specificity and off‐target effects of the sgRNA. The efficiency of the homologous repair of the AID tag at the cut site is greater if the required homologous repair site is near the sgRNA cut site (O'Brien, Wilson, Burgio, & Bauer, 2019; Inui et al., 2014). Increased distance between the cut site and the start codon or the stop codon can create challenges when designing homology arms. For example, when inserting an N‐terminal tag and using a guide with a cut site upstream of the start codon, the downstream homology arm can only extend to the start codon. The upstream homology arm can either end at the cut site, removing the 5′ UTR from the resulting product, or continue to the start codon. If this homology arm has extensive homology with both sides of the cut site, then the cut may be repaired without proper insertion of the template. The same types of challenges occur with cut sites internal to the protein coding region, as well as with C‐terminal tagging. However, silent mutations can be introduced into the homology arms to decrease sequence homology within the protein coding region without reestablishing a sgRNA recognition site.

As described in Basic Protocol 2, step 5, the sgRNA's proximity to the homologous recombination site takes priority over the specificity scores. Design at least three sgRNA to each terminus and clone into pX458 (Ran et al., 2013) (or another appropriate vector), which harbors GFP that can be used to quantify the efficiency of transfection. If an ideal sgRNA is not found, check the possibility of using other Cas9 enzymes with different PAM sequence requirements (Kleinstiver et al., 2015). Guide RNAs must be designed with the 3′ PAM sequence but do not include the PAM sequence in the cloned sgRNA construct.

The U6 promoter in the pSpCas9(BB)‐2A‐GFP (pX458) plasmid requires a “G” nucleotide at the beginning of the guide to efficiently transcribe the sgRNA. If the sgRNA designed does not have a “G” at the 5 end, add one “G” 5′ of the forward sequence of the guide RNA and the reverse complement of the “G” in the reverse sequence.

#### Homology‐directed repair

When using PCR products as the HDR template, two phosphorothioate moieties are added to the first two 5′ nucleotides of each primer to increase PCR product stability in the cell (Zheng et al., 2014). Homology arms can vary in length from 800 nt to <10 nt (Lambrus et al., 2018; Paix et al., 2017; Sakuma, Nakade, Sakane, Suzuki, & Yamamoto, 2016). We recommend 50‐nt homology arms on both sides of the cut site for the AID integration (Sathyan et al., 2019).

#### Designing primers for the C‐terminal HDR construct

The 3′ UTR can be critical in regulating gene expression, and any changes in the sequence could modulate expression levels. If possible, select sgRNAs that cut inside the gene prior to the stop codon. In the C‐terminal region, if the cut site is before the stop codon, you should include wobble substitutions for C‐terminal amino acids in the forward primer. Wobble substitutions proximal to the Cas9 cleavage site have a two‐fold purpose: (1) they ensure that the only homologous region in the donor is upstream of the cut site and (2) they prevent reconstitution of the sgRNA recognition site.

If no appropriate sgRNAs cut prior to the stop codon, then you should choose the sgRNA that cuts downstream and closest to the stop codon. This reduces the challenges affecting the regulatory elements of the gene while designing the repair construct.

In all cases in which the desired homologous recombination event recreates the original guide sequence with fewer than two mismatches and an intact PAM sequence, mutate the relevant homology arm to abrogate guide binding. Use a silent mutation to destroy the PAM sequence if possible, or use two silent mutations near the 3′ end of the guide sequence (Cong et al., 2013). Check the evolutionary conservation of the wobble nucleotides (Ramani, Krumholz, Huang, & Siepel, 2019) to prioritize less conserved nucleotides. Check a codon usage chart to prioritize codons used at a similar frequency to the replaced codon (Athey et al., 2017).

#### Designing primers for the N‐terminal HDR construct

For N‐terminal tagging, if the cut site is after the start codon, you should include wobble substitutions for N‐terminal amino acids in the reverse primer. The 5′ UTR can be important in the regulation of gene expression; therefore, any changes in the sequence should be avoided. Select sgRNAs that cut inside the gene after or very proximal to the start codon.

### Troubleshooting

#### Choosing to tag the N or C terminus

The addition of any tag to the N‐terminal or C‐terminal of a protein could disrupt its function. Therefore, the functionality of N and C‐terminally AID‐tagged proteins should be empirically determined. For this reason, we recommend initially tagging each end of the protein independently.

#### Lack of positive colonies or too many colonies during tagging

If there is difficulty in getting positive colonies, check the efficiency of the sgRNA using the Surveyor assay and try a different sgRNA if efficiency is low. Another potential problem is the disruption of the protein function by tagging with the AID. If tagging disrupts the function of a haploinsufficient gene or if tagging generates a dominant negative mutant, then you will not get positive colonies. The rate of homozygous integration is ∼10% of the heterozygous integration in HEK293T cells, which have hyperdiploid chromosome numbers.

If tagging with AID does not result in colony growth, this may be due to low expression of the gene of interest. Note that the antibiotic selection marker (*HygR*) is cotranscribed with AID and the protein products are separated during translation. Therefore, the resistance marker will be expressed at levels comparable to the target protein.

If there are too many colonies and it is difficult to pick individual colonies, then split cells and plate ∼100 to 200 cells per 10‐cm plate. Depending on the cell type and cell survival after splitting, change the number of seeded cells. Grow cells with conditioned medium to help individual cells to form colonies. Approximately 50 colonies in a 10‐cm plate is optimal.

#### Testing the functionality of the tagged proteins

Absence of any tagged colonies may indicate that the tagged protein is not functional. If you are not able to generate homozygous clones after screening several clones (close to 100 heterozygous clones), then attempt to tag the protein at the other terminus. We recommend tagging a gene such as *ZNF143* as a positive control, as *ZNF143* is ubiquitously expressed and we previously optimized these sgRNAs and confirmed that C‐terminally tagged *ZNF143* is functional (Sathyan et al., 2019). To test the functionality of the tagged proteins, initially look at whether the protein localizes to the same compartment as the untagged proteins using immunofluorescence or cell fractionation. The same localization may indicate the protein is functional. For transcription factors, genomic localization is an indication of functional transcription factor binding, and quantitative chromatin immunoprecipitation and sequencing (ChIP‐seq) can be used to determine whether degradation results in genome‐wide unidirectional decreases in binding (Guertin, Cullen, Markowetz, & Holding, 2018). Check the proximity of transcription factor binding relative to the genes that change expression upon auxin treatment; the class of activated or repressed genes is expected to be, on average, closer to the transcription factor's binding sites compared to the unchanged gene class. Depending upon the function of the protein, query the appropriate molecular phenotypes to confirm auxin‐induced deficiencies.

#### Little or no degradation upon auxin treatment

Although the AID system works in many cell types and organisms, each cell type and organism is unique, and the cofactors of the ubiquitin system may be differentially active. Check the expression of the components of the endogenous ubiquitin system and ensure that they are expressed.

The ARF‐AID system requires full‐length AID (Fig. 6). ARF interacts with domains III and IV of AID. Domains I and II are involved in the interaction with TIR1. The mini‐AID lacks domains III and IV and will not interact with ARF to stabilize the protein in the absence of auxin (Sathyan et al., 2019).

#### Activating the aryl hydrocarbon receptor

A caveat when using any auxin system is that the auxins are aromatic hydrocarbon molecules, and indole‐3‐acetic acid (auxin) can cause changes in expression of aryl hydrocarbon receptor target genes (Sathyan et al., 2019). Using proper negative controls and filtering out the aryl‐hydrocarbon‐receptor‐regulated genes alleviates this problem.

### Understanding Results

#### Insertion of ARF‐TIR1 into the AAVS1 locus

The forward and reverse primers flank the genomic DNA integration site of the ARF‐TIR1 construct (Fig. 2A). If there is no insert, the PCR produces a smaller product with the flanking primer, whereas it yields a larger product if ARF‐TIR1 is properly integrated. Heterozygous integration of the construct results in the two bands. Independently, we also perform a PCR using the flanking forward primer and a primer that is internal to the insert to confirm the integration of the construct at the locus (Fig. 2B).

#### Interpreting the Surveyor assay

If the sgRNA cutting site is at the center of the PCR product, the two cannot be resolved, whereas unequal‐sized fragments run as distinct bands. There is no need to mix wild‐type PCR products with the PCR products of the sgRNA‐transfected cells for the assay because many different repair products form and many sites in the population will remain unmodified.

#### Interpreting PCR screening for successful AID tagging

Successful integration of the AID‐P2A‐HygR or HygR‐P2A‐AID results in an addition of ∼1785 bp to the PCR product. Heterozygous clones will have two bands, one with a length of genomic region between forward and reverse primer and the second with a length of genomic region between primers plus the 1785 bp. The homozygous integration will result in one band with a length of genomic region between primers plus the 1785 bp. Sequence the integrated DNA using the same forward primer, which will confirm successful integration.

### Time Considerations

There are two components in the canonical AID, ARF‐AID, and AID‐ARF clamp systems, the generation of a progenitor cell and tagging the protein of interest with the degron. A general outline of the timeline to complete each step for HEK293T cells is given below, which may vary between cell lines used.

ARF‐TIR1 or TIR1 progenitor line: 6‐8 weeks*

Design, clone, and test sgRNAs: 4 weeks*

Design, and order the primers and PCR HDR constructs: 1 week*

Tag the gene of interest with AID tag: 6‐8 weeks.

Steps marked * can be completed simultaneously.
